# The negative affect repair questionnaire: factor analysis and psychometric evaluation in three samples

**DOI:** 10.1186/1471-244X-13-16

**Published:** 2013-01-09

**Authors:** Anne Scherer, Nicole Eberle, Maren Boecker, Claus Vögele, Siegfried Gauggel, Thomas Forkmann

**Affiliations:** 1 Institute of Medical Psychology and Medical Sociology, University Hospital of RWTH Aachen, Pauwelsstraße 19, Aachen, 52074, Germany; 2 Research Group Self-Regulation and Health, Research Unit INSIDE, University of Luxembourg, Route de Diekirch - B.P. 2, Walferdange, 7220, Luxembourg, Luxembourg

**Keywords:** Affect regulation, Emotion regulation, Mood regulation, Psychometric quality

## Abstract

**Background:**

Negative affect and difficulties in its regulation have been connected to several adverse psychological consequences. While several questionnaires exist, it would be important to have a theory-based measure that includes clinically relevant items and shows good psychometric properties in healthy and patient samples. This study aims at developing such a questionnaire, combining the two Gross [1] scales Reappraisal and Suppression with an additional response-focused scale called Externalizing Behavioral Strategies covering clinically relevant items.

**Methods:**

The samples consisted of 684 students (mean age = 23.3, *SD* = 3.5; 53.6% female) and 369 persons with mixed mental disorders (mean age = 36.0 *SD* = 14.6; 71.2% female). Items for the questionnaire were derived from existing questionnaires and additional items were formulated based on suggestions by clinical experts. All items start with “When I don’t feel well, in order to feel better…”. Participants rated how frequently they used each strategy on a 5-point Likert scale. Confirmatory Factor Analyses were conducted to verify the factor structure in two separate student samples and a clinical sample. Group comparisons and correlations with other questionnaires were calculated to ensure validity.

**Results:**

After modification, the CFA showed good model fit in all three samples. Reliability scores (Cronbach’s α) for the three NARQ scales ranged between .71 and .80. Comparisons between students and persons with mental disorders showed the postulated relationships, as did comparisons between male and female students and persons with or without Borderline Personality Disorder. Correlations with other questionnaires suggest the NARQ’s construct validity.

**Conclusions:**

The results indicate that the NARQ is a psychometrically sound and reliable measure with practical use for therapy planning and tracking of treatment outcome across time. We advocate the integration of the new response-focused strategy in the Gross’s model of emotion regulation.

## Background

Negative affect has profound effect on the quality of social interactions, social functioning, and well-being [1-4]. Consequently, people try to regulate negative affect using various strategies, with some strategies apparently being more successful than others [4,5]. Difficulties in affect regulation are considered to be connected to several adverse consequences, from ordinary unhappiness to outright psychopathology [6,7], such as mood disorders [8], generalized anxiety disorder [9], personality disorders [10] and substance abuse [11,12].

Nonetheless, some problems come to light when discussing affect regulation. For example, the terms affect, emotion, and mood are used inconsistently, often interchangeably. In order to avoid this confusion, we use the term “affect regulation” in the sense of a superordinate category for all valenced states. Consequently, negative affect regulation describes the tendency to actively and positively influence negative affect with various strategies. This includes the regulation of emotions and moods.

Another problem concerns the available instruments to measure affect regulation. Many instruments are purely empirically derived (by use of exploratory factor analyses), i.e., without clear theoretical foundation or without testing the appropriateness of the theoretical model used. This may lead to uncertainties about the number and interpretation of the empirically derived factors [13,14]. Some of these instruments include Thayer et al’s questionnaire [5] or Garnefeski at al’s *Cognitive Emotion Regulation Questionnaire* (CERQ), where a principal component analysis was used [15]. Other measurement instruments assess affect regulation as one aspect embedded in a broader theoretical construct, for example emotional intelligence or generalized expectancies of negative mood regulation [16-18]. Several more instruments to assess emotion or mood regulation strategies exist, especially in the context of certain therapeutic outcomes, for example, the Acceptance and Action Questionnaire (AAQ) [19], which was developed in connection with Acceptance and Commitment Therapy (ACT) [20], or the Emotion Regulation Skills Questionnaire [21] which asks participants about their view on different competencies in relation with emotion regulation and which is connected to an emotion regulation training [22]. Diverse coping scales also include items similar to those found in affect regulation literature.

Despite its extensive use in clinical application, most available assessment tools of affect regulation strategies were developed exclusively with student populations. This is probably a critical point, making their use potentially difficult for clinical groups (*Negative Mood Regulation* (NMR) [16]; *Difficulties in Emotion Regulation Scale* (DERS) [17]). In the closely related field of coping assessment, Parker et al. [23] could not replicate the factor structure of a well-established coping questionnaire in different student samples, proving the complexity of developing an assessment tool with stable psychometric properties across different samples.

With a plethora of different instruments used in empirical studies, it is not surprising that the last years saw a rise of different taxonomies of affect regulation. For a comprehensive overview, see the recent meta-analyses by Webb et al. [24] and Aldao et al. [25] and the recent review by Berking and Wupperman [26]. Of the theories available in the field of affect regulation, Gross’s Emotion Regulation Theory is probably one of the most intensively investigated and most widely accepted [1]. Gross's model includes five distinct emotion regulation stages, which occur in sequence over time. The strategies of the first four stages are summed up under the term “antecedent-focused strategies”, because they are assumed to be used before the emotion takes (full) hold. So antecedent-focused emotion regulation means people try to change the way they approach an emotion-eliciting situation. The sequence starts with Situation Selection and Situation Modification. After these two steps follows Attentional Deployment and finally, Cognitive Reappraisal of the emotion-eliciting situation.

In contrast, response-focused strategies are employed to change an already existing emotion in certain ways. The only strategy of the response-focused stage explicitly stated in Gross’s model is “Expressive Suppression”. This strategy is defined by attempting to keep emotions in check by not showing them, especially toward other people. Reappraisal, unlike Suppression, is supposed to ameliorate the effects that negative emotions have on the person. Suppression, in fact, may even be detrimental to well-being by making people feel inauthentic [27] and causing disruptions in social situations [28].

Over the past fifteen years, much evidence has been presented supporting the general validity of the model (e.g., [28,29]), while on the other hand, there is so far little evidence concerning the sequence of different time stages, the allocation of the strategies to a certain stage and the question if antecedent-focused strategies are generally more beneficial than response-focused strategies. For a more in-depth discussion on these issues, see [26].

Nevertheless, a close link of the postulated emotion regulation strategies Reappraisal and Suppression to personal well-being and the development and maintenance of mental disorders has been shown (e.g., [15,27,30-32]).

Starting from Gross’ model, Gross and John developed the *Emotion Regulation Questionnaire* (ERQ; [27]), which was designed to measure Reappraisal and Suppression - the two emotion regulation strategies the authors considered to be most typical. In their original study, Gross and John found internal consistencies ranging from .68 to .82 with no cross-loadings on the two factors [27]. Egloff et al. [33] reported good internal consistencies (mean α = .81) for a German adaptation of the ERQ in a sample of 82 psychology students. While the ERQ includes regulation of positive and negative emotions, no separation is made between regulation of positive and negative emotion in the sum score, assuming that the valence of the regulated emotion is of less importance than the strategy in general. The wording of the items are very similar, asking for Reappraisal and Suppression on an abstract level and with seven answer options, which might be difficult to answer and to interpret, especially for people with mental disorders [34]. Additionally, the factor structure of the ERQ could not be replicated by Stadelmaier [35].

From a clinical perspective, it might be surprising that other strategies to reduce unpleasant feelings are still absent from Gross’s model and thus also from the ERQ. Self-harm, aggression or substance abuse should also be counted among the response-focused emotion regulation strategies because they are usually used when the negative affect already took hold. These strategies could be taken together as “Externalizing Behavioral Strategies” and should be added as additional response-focused strategy to Gross’s model, especially because knowing about strategies which are harmful to the person’s health at the beginning of treatment can be crucial to its success [36]. A typical patient sample expected to show several harmful externalizing behavioral strategies are people diagnosed with Borderline Personality Disorder (BPD). In BPD, potentially self-damaging impulsivity in at least two areas and inappropriate anger are diagnostic criteria according to DSM-IV [37]. Patients suffering from BPD are likely to drop out of inpatient [38] or outpatient treatment [39] and have a high risk of completed suicide [40]. But of course, externalizing behavior is also common in other mental disorders without a necessary comorbidity of a personality disorder [41].

Externalizing behavioral strategies of affect regulation are not usually covered by affect regulation questionnaires. The questionnaire developed by Thayer et al. [5] assesses the use of drugs and alcohol with two items. Another questionnaire was developed by Phillips and Power [42] to monitor functional and dysfunctional emotion regulation strategies in adolescents and single items concerning externalizing behavior exist in several affect regulation and coping questionnaires. Nevertheless, to our knowledge, no other questionnaire assesses the strategies of self-harm, aggression, substance abuse or other externalizing behavioral strategies to regulate negative affect in a systematic manner.

To sum up, there is extensive evidence suggesting that affect regulation may play an important role in the development and maintenance of mental disorders and that the two extensively investigated regulation strategies Cognitive Reappraisal and Expressive Suppression are related to personal well-being. However, from a clinical perspective, it appears promising to extend Gross’s model of emotion regulation by a second response-focused strategy called “Externalizing Behavioral Strategies” which appears to be of major importance in clinical populations and thus also promises important practical benefits. To our knowledge there is no instrument that combines the two Gross scales with an assessment of Externalizing Behavioral Strategies. This provided the rationale for the current study which aimed at developing the Negative Affect Repair Questionnaire (NARQ) including those three scales, measuring them with more palpable, behavior-related items that measure different aspects of Reappraisal and Suppression and ensuring their stability across clinical and non-clinical groups. The focus on negative affect allows us to exclude confounding effects that could arise from differences in regulation of positive and negative emotions. Consistent with previous results and Gross’s theoretical model, we assumed the questionnaire to have a three-dimensional structure. Furthermore, we expected Reappraisal to be related to psychological well-being and Suppression and Externalizing Behavior to be related to symptoms of mental illness. We also expected general differences in the emotion regulation strategies between men and women as found by Gross and John in their original work on the ERQ [27] as well as Nolen-Hoeksema & Rusting [43] and differences in Externalizing Behavior between patients with and without a diagnosis of Borderline Personality Disorder.

## Methods

### Participants

The study included 684 students, most of whom studied medicine, psychology or engineering, and 369 persons who were treated at a local psychotherapeutic hospital. Students’ mean age was 23.3 years (*SD* = 3.5), 53.6% were female. Mean age of people diagnosed with mental disorders was 36.0 years (*SD* =14.6), 71.2% were female. The most common primary diagnoses were Major Depressive Disorder (40.4%) and Eating Disorders (29.3%). Diagnoses were verified in a two step procedure: First, the diagnoses were assessed by the responsible therapist using a clinical interview in which the International Diagnostic Checklists (IDCL) [44] were applied. The IDCL are checklists that can be used to make a careful evaluation of the symptoms and classification criteria, and thus help to arrive at precise diagnoses according to ICD-10 criteria. Results of this assessment were discussed with a supervising senior psychotherapist. In case that ambiguity about main and comorbid diagnoses persisted after this it was decided that a full form of the German Version of the Structured Clinical Interview for DSM-IV (SKID) [45] was conducted in addition. The clinical interviews were conducted by clinical psychologists. In the second step, diagnoses were verified through clinical conferences including senior psychotherapists and psychiatrists. Table 1 shows detailed demographic and clinical characteristics of the samples. The students were randomly divided into two subsamples to allow for cross validation. The two random groups did not differ in age (*t* = 1.34, p = .184) or sex (*χ*^*2*^ =1.42, p = .251). Students volunteered to participate after class meetings. Persons seeking treatment for mental disorders were tested during admission as part of a standard diagnostic assessment. They gave written informed consent for their data to be included in research. Ethical approval was given by the local ethics committee of the Medical Faculty of the RWTH Aachen University. None of the participants was paid.

**Table 1:** Demographic and clinical characteristics of the student and clinical groups and the total sample

	**N**	**Age**			**Female %**	**Primary diagnoses**		
		**M**	**SD**	**Range**		**Depression**	**Eating disorder**	**Other**
**Total sample**	1053	27.7	11.0	15 - 81	59.8			
**Students I**	341	23.4	3.7	19 - 45	51.3			
**Students II**	343	23.0	3.4	18 - 50	55.8			
**Clinical**	369	36.0	14.6	15 - 81	71.2	40.4%	29.3%	30.3%

### Instruments

#### Item pool

Items designed to assess strategies that people might use to repair negative affect were collected. The item pool was constructed as follows: questionnaires related to affect regulation were screened to select relevant items, some of which were revised or rephrased to standardize wording and answer options. Additional items related to behavior and relevant for clinical populations were constructed. For the second step, clinicians (i.e., senior psychotherapists) were asked to suggest further relevant regulation strategies, especially those they rated as clinically relevant. The final item pool consisted of 55 items. All items start with “When I don’t feel well, in order to feel better…”. Test subjects rated how frequently they used each of the strategies on a 5-point Likert scale ranging from 0 (never) to 4 (always).

Consistent with previous results and Gross’s theoretical model, we assumed the questionnaire to have a three-dimensional structure. Items were allocated to one of the three scales—Reappraisal, Suppression, or Externalizing Behavioral Strategies. Items that could not be allocated to one of the three categories were excluded. Thus, analyses were performed using 32 items.

#### Emotion regulation questionnaire

The Emotion Regulation Questionnaire. (ERQ, [27]) consists of 10 items (e.g., I keep positive emotions to myself; I control my negative emotions by not expressing them.). Six of them are included on the “Cognitive Reappraisal” scale, four on the “Expressive Suppression” scale. Answers are given on a scale that ranges from 1 “strongly disagree” to 7 “strongly agree”, which adds up to a minimum score of 6 (Reappraisal) and 4 (Suppression) and a maximum score of 42 (Reappraisal) and 28 (Suppression). The ERQ was completed by the sample of persons with mental disorders only.

#### Beck depression inventory

The Beck Depression Inventory (BDI, [46,47]) was used to measure depression. It contains 21 items answered on a 0-to-3 scale. Participants were asked to choose one or more statements per item that best represented their mental state during the last week. A total score of ≥11 indicates mild-to-moderate depression, and a total score of ≥18 indicates moderate-to-severe depression. The BDI was completed by the sample of persons with mental disorders only. Internal consistency (Cronbach’s α) for the BDI was .90 in our sample.

### Data analysis

#### Confirmatory factor analysis

Confirmatory factor analysis (CFA) was conducted to corroborate the postulated factor structure of the NARQ and to determine its reliability. Ordinal scale CFA with weighted least square means and variance adjusted was performed using the software MPlus 4.1. The appropriateness of a specific CFA model was assessed using absolute and incremental measures of global model fit. Measures of global fit indicate whether the empirical associations among the manifest variables are appropriately reproduced in the model [48,49].

Absolute fit was tested with two measures: *χ*^2^ is used to measure the differences between the observed and the expected covariance matrix. But because *χ*^2^ is known to be sensitive to sample size, additional measures were included [50,51].

The root mean square error of approximation (RMSEA) is interpreted as the amount of information within the empirical covariance matrix that cannot be explained by the proposed model. The model may be classified as acceptable if only 8 percent or less of the information is not accounted for by the model (RMSEA ≤0.08; [51]).

Measures of incremental fit show the improvement of the proposed model compared with a baseline model. Two different measures of incremental fit were employed in this study: The Tucker-Lewis index (TLI) and the Comparative Fit Index (CFI) [52]. TLI and CFI values of ≥ .95 indicate a good model fit [53].

To improve fit, (1) items were eliminated if item-scale correlations were low (<.32; [53]) and the elimination of the item would not reduce the internal consistency of the scale, or (2) modification indices were allowed when they suggested that residual correlations between error terms would entail a substantial improvement in fit, a residual correlation was in line with theoretical considerations and the modification could be replicated in the cross-validation [49,54].

All analyses were first conducted using one of the student samples and then cross-validated on the second student sample and the sample of persons with mental disorders.

Additionally, the factor structure of the ERQ was tested in the clinical sample to compare its factor structure with our newly developed questionnaire.

#### Reliability and validity

Reliability (internal consistency) of the three scales of the NARQ was assessed by calculating Cronbach’s α. Validity was assessed by using Multivariate Analysis of Variance (MANOVA) and effect sizes to compare mean values for subgroups that were expected to differ in affect regulation. Effect sizes *d* were calculated using Hedges and Olkin’s corrected factor [55]. According to Cohen’s guidelines, effect sizes of .20 < *d* ≤ .50 were interpreted as small, .50 < *d* ≤ .80 as medium, and d *≥* .80 as large [56]. Students were compared to persons with mental disorders, female students to male students and persons with a diagnosis of Borderline Personality Disorder to persons with other diagnoses.

Students and persons with mental disorders were compared because students were expected to use more Reappraisal, but less Suppression and Externalizing Behavioral Strategies [57]. Male and female students were compared because women were expected to use less Suppression and less Externalizing Behavioral Strategies but not to differ from men in Reappraisal [27,43]. Persons with a diagnosis of Borderline Personality Disorder were compared to people with other diagnoses because the former were expected to use more Externalizing Behavioral Strategies than the latter [58,59].

To assess construct validity, scores on the final version of the NARQ were compared to the scores of the ERQ scales, expecting significant, but not perfect correlations and the BDI, expecting a small negative correlation with the Reappraisal scale and a small positive correlation with the Suppression scale. A higher positive correlation was expected between the BDI and Externalizing Behavior Strategies because of the higher clinical relevance of the scale. All correlations were calculated using Pearson’s *r*. All reliability and validity calculations were performed in SPSS 18.

## Results

### Confirmatory factor analysis

Confirmatory Factor Analysis (CFA) was initially performed using 32 items as indicators of the underlying three latent constructs (Reappraisal, Suppression, and Externalizing Behavioral Strategies). According to global-fit measures, the original CFA model did not show a good fit to the data (*χ*^2^ = 1697.0, p < .001, RMSEA = .112, TLI = .72, CFI = .72). None of the fit criteria was in the acceptable range; therefore, 15 items were removed according to the guidelines (see Methods). In addition, modification indices indicated that several items that loaded on the same factors should be allowed to have correlated error terms. Thus residual correlations that were in line with theoretical considerations were allowed. The modified CFA model that resulted contained 17 items and yielded a better fit of the data (*χ*^2^ = 1598.9, p < .001, RMSEA = .064, TLI = .96, CFI = .96) that was confirmed in all three samples (see Table 2).

**Table 2:** Measures of global fit for all models estimated and Hierarchical model tests

	***χ***^**2**^	***df***	***p***	**TLI**	**CFI**	**RMSEA**
Thresholds for acceptable fit			> .05	≥ .90	≥ .90	≤ .08
**First Version**						
Students I	1697.0	99	<.001	.72	.72	.112
**Modified Version**						
Students I	1598.9	46	<.001	.96	.96	.064
Students II	1122.7	43	<.001	.93	.94	.067
Clinical	1915.2	38	<.001	.96	.95	.074

**Figure 1:**
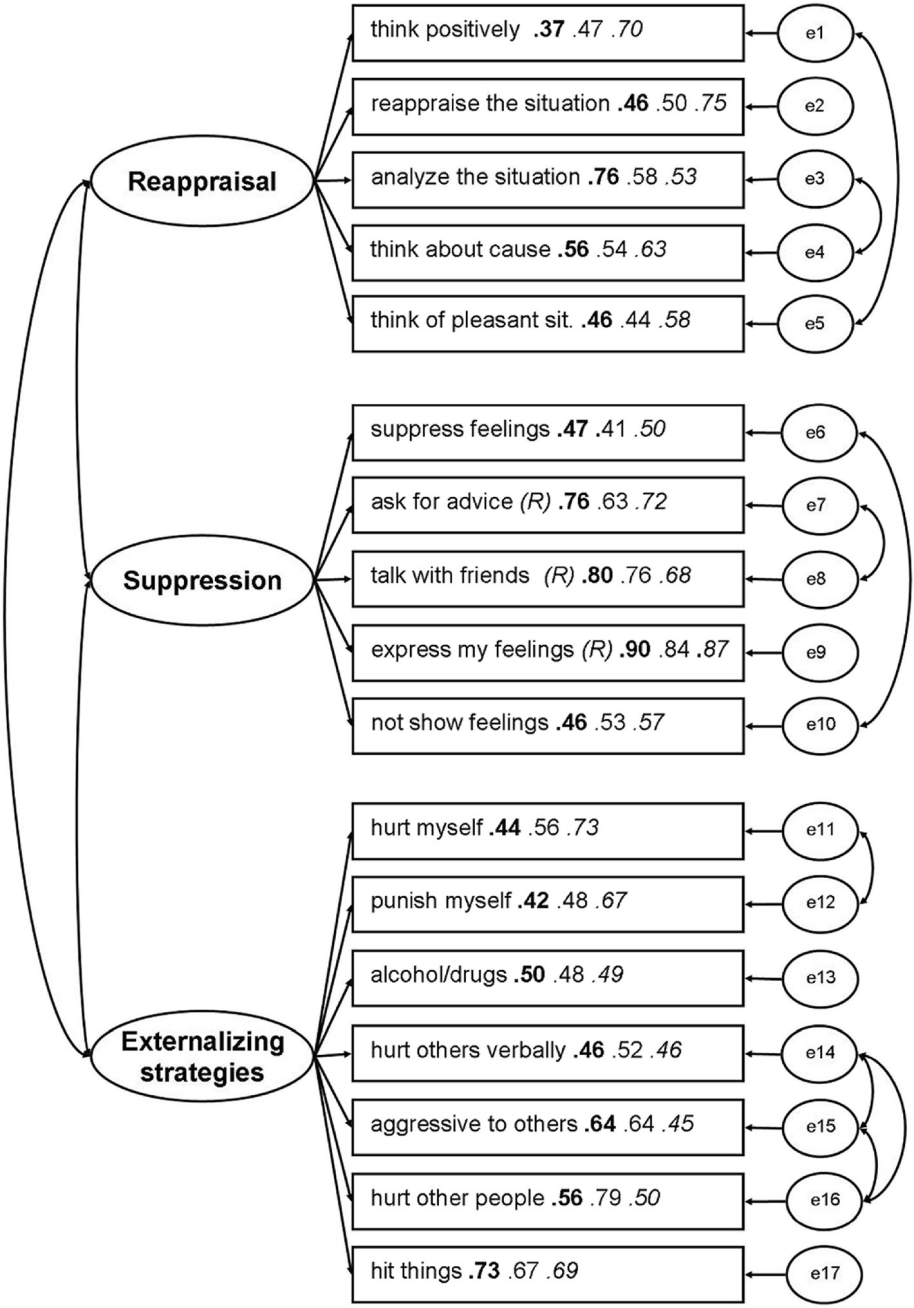
**Structure of the CFA model.** Note: Bold numbers = First student sample, normal numbers = Second student sample, italic numbers = Clinical sample

The item-scale correlations for each item and the scale structure of the modified CFA model are displayed in Figure 1, means and standard deviations for students and clinical subgroups are displayed in Table 3. All items of the final version of the NARQ scales can be found in the Additional file 1.

**Table 3:** Means and standard deviations of student sample and clinical subgroups

	**N**	**Reappraisal**			**Suppression**			**Externalizing Behavior Strategies**		
		**M**	**SD**	**Range**	**M**	**SD**	**Range**	**M**	**SD**	**Range**
**Students**	684	11.4	3.0	1 – 20	8.6	3.7	0 – 20	3.9	3.2	0 – 19
**Clinical: Depression**	147	9.1	3.5	0 – 17	10.7	4.0	1 – 20	4.6	3.7	0 – 14
**Clinical: Eating Disorders**	107	8.7	3.5	1 – 18	10.9	3.6	2 – 17	5.6	3.8	0 – 18
**Clinical: Other**	115	10.0	3.0	3 – 20	10.8	3.6	1 – 18	4.8	4.1	0 – 18

Note: Clinical sample sorted by first diagnosis. Further comorbid disorders may exist.

Additionally, we tested the ERQ’s assumed theoretical factor structure for the clinical population. While TLI and CFI showed good results (.96 and .95 respectively), the RMSEA was .92, indicating insufficient global fit. One of the reappraisal items fell below the factor loading of .32.

### Pearson’s intercorrelations between NARQ scales

Analyses were conducted to examine the relationship between the three NARQ scales. Results revealed significant but low correlations between all scales: There were negative correlations between Reappraisal and Suppression (*r* = −.34, *p* < .001), and between Reappraisal and Externalizing Behavioral Strategies *(r* = −.24, *p* < .001). A positive correlation was found between Suppression and Externalizing Behavioral Strategies *(r* = .18, *p* < .001).

### Reliability and validity of the NARQ

In order to determine the reliability of the NARQ, internal consistencies of the theoretically and empirically derived scales were calculated. The total sample was used for this analysis. The internal consistencies for the NARQ, structure based on the modified CFA model, were satisfactory: α = .73 for Reappraisal (5 items), α = .80 for Suppression (5 items) and α = .71 for Externalizing Behavioral Strategies (7 items).

Scale means and standard deviations for the student population were *M* = 11.4 (*SD* = 3.0) for Reappraisal (max. 20), *M* = 8.6 (*SD* = 3.6) for Suppression (max. 20) and *M* = 3.9 (*SD* = 3.2) for Externalizing Behavioral Strategies (max. 35). Scale means and standard deviations for the clinical sample were *M* = 9.3 (*SD* = 3.4) for Reappraisal, *M* = 10.7 (*SD* = 3.8) for Suppression and *M* = 4.9 (*SD* = 3.9) for Externalizing Behavioral Strategies.

In both subgroups (students and patients), Reappraisal and Suppression were the most commonly used strategies. Calculating effect sizes between the two samples, students use more Reappraisal (*F* = 101.46 *p* < .001; *d* = .68 CI = .55 to .81), but less Suppression (*F* = 81.19 *p* < 0.001; *d* = −.59 CI = −.46 to -.72) and Externalizing Behavioral Strategies (*F* = 17.10 *p* < .001; *d* = −.29 CI = −.16 to -.42) than patients. Comparing male and female students, male students showed slightly less Reappraisal (*F* = 9.94 *p* = .002; *d* = .25 CI = .09 to .40), but markedly more Suppression and Externalizing Behavioral Strategies (*F* = 101.46 *p* < .001; *d* = −.81 CI = −.65 to -.97, *F* = 31.00 *p* < .001; *d* = −.44 CI = −.29 to -.60) than female students.

Persons diagnosed with Borderline Personality Disorder showed Reappraisal and Suppression no different from persons with other mental disorders (*F* = .39 *p* = .561; *d* = .13 CI -.19 to .45; *F* = 2.60 *p* = .108; *d* = −.31 CI .01 to -.63). On Externalizing Behavioral Strategies, on the other hand, persons with BPD reached twice the raw score of other people, resulting in a large effect size (*F* = 65.76 *p* < .001; *d* = −1.33 CI −1.00 to −1.66). See Table 4 for details on means and standard deviations.

**Table 4:** Means and standard deviations for the different subgroups

	**Reappraisal**			**Suppression**			**Externalizing**		
	***N***	***Mean (SD)***	***d (CI)***	***N***	***Mean (SD)***	***d (CI)***	***N***	***Mean (SD)***	***d (CI)***
**All Persons with Mental Disorders**	364	9.3 (3.4)	0.68 (0.55- 0.81)	364	10.7 (3.8)	−0.59 (−0.46 − -0.72)	350	4.9 (3.9)	−0.29 (−0.16- −0.42)
**All Students**	670	11.4 (3.0)		675	8.6 (3.6)		673	3.9 (3.2)	
**Male Students**	314	11.0 (3.2)	0.25 (0.09-0.40)	312	10.0 (3.3)	−0.81 (−0.65 − -0.97)	312	4.7 (3.5)	−0.44 (−0.29 − -0.60)
**Female Students**	356	11.8 (2.8)		363	7.3 (3.4)		361	3.3 (2.8)	
**BPD**	43	8.9 (3.4)	0.13 (−0.19-0.45)	43	11.8 (3.7)	−0.31 (0.01 − -0.63)	44	9.0 (3.7)	−1.33 (−1.00 − -1.66)
**Other Mental Disorders**	318	9.3 (3.4)		319	10.6 (3.8)		304	4.3 (3.5)	

Note: All comparisons except for Reappraisal and Suppression between BPD and other patients were significant (p < .001).

In order to explore construct validity, correlations between the NARQ scales and BDI scores were assessed for the clinical subgroup. The results revealed a weak but significant negative correlation between the BDI and NARQ scale Reappraisal (*r* = −.35, *p* < .001), indicating that an increased score on the Reappraisal scale of the NARQ is associated with fewer depressive symptoms.

In contrast, the correlations between the scales Suppression and Externalizing Behavioral Strategies and the BDI score were positive (*r* = .36, *p* < .001 and *r* = .49, *p* < .001) suggesting that with an increased score in Suppression and Externalizing Behavioral Strategies, the depression score also increased. Comparing the correlations to the correlations between the BDI and the original ERQ scales, we found an identical correlation for the Suppression Scale (*r* = .36, *p* < .001), but a lower correlation for the ERQ Reappraisal Scale (*r* = .20, *p* = .004).

Correlations of the NARQ scales with the ERQ scales could also only be obtained from the clinical sample and were calculated to estimate how closely the NARQ scales match the original scales by Gross and John [15]. The results show moderate correlations between the Reappraisal scales (*r* = .52, *p* < .001) and the Suppression scales (*r* = .72, *p* < .001). Weaker but significant correlations were also found between the ERQ scale Reappraisal and the NARQ scale Suppression *r* = −.18, *p =* .006 and between the ERQ scale Suppression and the NARQ scales Reappraisal *r* = −.29, *p* < .001 and Externalizing Behavioral Strategies *r* = −.22, *p* = .001.

Summing up, the results show acceptable fit for the modified model and indicate good internal consistency and construct validity of the new questionnaire.

## Discussion

The aim of the current study was to develop a theoretically derived and reliable instrument for the measurement of negative affect repair strategies, applicable in clinical groups and assessing the well established scales Reappraisal and Suppression in addition to a scale measuring Externalizing Behavioral Strategies that may endanger the therapeutic process. The psychometric properties of the newly developed NARQ were examined in two student samples and a clinical sample.

The a-priori, theoretically derived scale structure was tested using CFA. After modification and shortening the scale to 17 items, the three-dimensional structure could be found in the student sample and confirmed in the second student sample and the clinical sample. The results are in line with the hypothesized three-dimensional factor structure of the NARQ and confirm the general multidimensionality of the construct negative affect regulation [5,17,27,60].

Reliability was found to be acceptable, especially in regard to other published affect regulation questionnaires, for example the ERQ (α = .68 - .82), the questionnaire published by Thayer [5] (α = .54 to .81) or Phillips’s and Power’s questionnaire [42] (α = .66-.76).

Comparisons between clinical and student samples, between male and female students and between persons with BPD and persons with other mental disorders showed results that are in line with the theoretical expectations and thus can be counted as evidence for the NARQ’s validity. The comparably small difference between the clinical and the student sample in average use of externalizing strategies is not unexpected: Only a few patients show aggressive behavior (towards themselves or others) or drug abuse. Comparing persons with and without a diagnosis of BPD, larger differences came to light. The correlations with related and diverging constructs (BDI, ERQ) also supported the NARQ’s construct validity.

Some limitations of the present study should be noted. Due to the nature of self-reports, it cannot be ruled out that social desirability could have had an influence on the rating of strategies that might have been considered socially undesirable (e.g., hurt others, hit things). However, socially undesirable strategies were reported in all samples. A further potential methodological shortcoming was the significant *χ*^*2*^ of the CFA models which might be interpreted as indicating insufficient fit of the model. However, it is a well-known statistical effect that the *χ*^*2*^-statistic becomes hypersensitive for small misspecifications of the model in large samples resulting in significant results [50,51]. Since the entire study sample consisted of N = 1053 individuals this was likely the case in the present study. In this case, it is recommended to examine further fit indices to identify real serious misfit. Since all further measures of model fit (RMSEA, CFI, TLI) indicated very good model fit, the significant *χ*^*2*^-statistic can be neglected.

The deletion of items and the inclusion of correlated error terms are two empirical strategies to improve fit to the model in CFA that might be interpreted as a contradiction towards a theory-driven approach. However, in this study, we followed an a-priori protocol and only allowed correlated error terms if there was a good theoretical explanation for both items to have a common variance beyond the scale to which they were allocated and remained within the framework of Gross’s theory. None of the residual correlations allowed threatened the theoretically assumed dimensionality of the developed instrument. Since the identified factor structure could be replicated across all three samples it can be considered to be stable despite of the application of modifications [54].

The items included in the Reappraisal scale indicate a wider understanding of the construct “Reappraisal” than the Reappraisal subscale used by Gross and John [27], which probably also causes the smaller correlation between those two scales in contrast to the Suppression scales. These more diverse cognitive items were retained because they showed the best psychometric fit and reliability. Since the item formulations are less abstract it can be expected that they are easier to understand. Furthermore, redundancy of item contents can be seen as potential shortcoming of the ERQ so that greater item diversity was an explicit aim in the development of the NARQ. This also applies to the NARQ Suppression scale, which includes items that cover aspects of (not) sharing feelings in addition to hiding them from others. While it has recently been argued that Suppression might not be used solely as an affect regulation strategy but also as a way to prevent negative evaluation of others [26], this potential problem was avoided by the instruction which asks specifically for things to do to make oneself feel better.

Some potentially harmful strategies (e.g., smoking or eating) had to be deleted from the final version of the NARQ due to insufficient fit. It is important to note that of course no questionnaire can replace a thorough case history to identify more idiosyncratic ways of aggression towards self and others (sexual behavior, binge eating, etc.).

Students were not screened for symptoms of mental disorder, so it must be assumed that a small part of the student populations suffers from mental illness. Nonetheless, the NARQ was able to differentiate between students and persons with mental disorders on the group-level. Differences between age groups could not yet be calculated because of the small age range of the current samples. Because of this and since students tend to be a fairly homogeneous group, it would be advisable to validate the factor structure in an additional representative, non clinical sample from the general population as well as compare other, larger groups of people with mental disorders.

A future aim should be to determine retest reliability and predictive validity of the inventory, for example the associations of NARQ scales with clinically relevant behavioral outcomes, to further corroborate the instruments validity and practical utility. Further analyses of convergent validity in comparison to other measures of affect regulation would also be useful. Most importantly, norms have to be developed for both sexes and maybe also for different age groups to permit standardized comparisons.

## Conclusion

With the NARQ, clinicians have a new, psychometrically sound and reliable measure for the assessment of affect regulation strategies. Initial validation of the instrument indicated promising results. The NARQ covers both Gross’s scales Reappraisal and Suppression which were operationalized in a more behavior-related manner. It also includes a new scale called Externalizing Behavioral Strategies. The reported differences between samples of persons with different mental disorders indicate that this scale might be of practical use for therapy planning and tracking of treatment outcome across time. We advocate for the integration of this new response-focused strategy in the Gross’s model of emotion regulation. In regard to psychotherapy, future research might be able to identify even more response-focused strategies, for example in relation to conceptions from Acceptance and Commitment Therapy [20] or Mindfulness-Based Cognitive Therapy [61] that might be of practical importance from a clinical perspective.

Deficits in affect regulation are considered to take a central role in the development and maintenance of mood disorders [8]. Therefore, a reliable and valid instrument capable of revealing dangerous behavior utilized for affect regulation early and tracking it across treatment can be crucial to the success of psychotherapy.

## Abbreviations

AAQ: Acceptance and action questionnaire; ACT: Acceptance and commitment therapy; BDI: Beck depression inventory; BPD: Borderline personality disorder; CFA: Confirmatory factor analysis; CFI: Comparative fit index; DERS: Difficulties in emotion regulation scale; DSM-IV: Diagnostic and statitical manual of mental disorders,4^th^ edition; ERQ: Emotion regulation questionnaire; ICD-10: International statistical classification of diseases and related health problems; IDCL: International diagnostic checklists; MANOVA: Multivariate analysis of variance; NARQ: Negative affect repair questionnaire; NMR: Negative mood regulation; RMSEA: Root mean square error of approximation; RWTH: Aachen Rheinisch-Westfälische Technische Hochschule Aachen; SKID: Structured clinical interview for DSM-IV; SPSS: Statistical package for the social sciences; TLI: Tucker-Lewis-index.

## Competing interests

The authors declare that they have no competing interests.

## Authors’ contributions

AS conceived the study and the design, conducted the statistical analyses and wrote the manuscript. NE helped conceiving the study, participated in its design and the data acquisition, and helped to draft the manuscript. MB participated in the design of the study and the statistical analysis. CV helped conceiving the study and its design and participated in data analysis and interpretation. SG has been involved in drafting and revising the manuscript, and coordinated the study and data acquisition. TF participated in the analysis and interpretation of the data and helped to draft the manuscript. All authors read and approved the final manuscript.

## Pre-publication history

The pre-publication history for this paper can be accessed here:

http://www.biomedcentral.com/1471-244X/13/16/prepub

## Supplementary Material

Additional file 1 Appendix. Click here for file
