# Supplementary material for: The negative affect repair questionnaire: factor analysis and psychometric evaluation in three samples
Source: BMC Psychiatry. 2013 Jan 9;13:16. doi: 10.1186/1471-244X-13-16 (PMC3558324; doi:10.1186/1471-244X-13-16)
Supplement: Additional file 1 — Appendix. [file 1471-244X-13-16-S1.doc]

**APPENDIX**

Items of the NARQ scales. Items are Likert-scaled with scale points 0 (never), 1 (rarely), 2 (occasionally), 3 (often), 4 (always).

**Scale - Reappraisal**

——————————————————————————————————

**When I don’t feel well, in order to feel better…**

… I try to think positively.

… I try to reappraise the situation.

… I analyze the situation to try to understand why I feel the way I do.

… I think about how to avoid people or things that caused me to feel bad.

… I think about pleasant situations from the past.

**Scale – Suppression** _____________________________________________________________________

**When I don’t feel well, in order to feel better…**

**…** I try to suppress my feelings.

… I consider asking other people for their advice. *(R)*

… I talk with my friends. *(R)*

… I express my feelings. *(R)*

… I do not show other people how bad I am feeling.

**Scale - Externalizing strategies**

——————————————————————————————————

**When I don’t feel well, in order to feel better…**

… I hurt myself.

… I punish myself.

… I drink alcohol or take some drugs to help me relax.

… I hurt others by the things I say

… I hurt other people

… I am aggressive towards other people

… I hit things

*(R)* = revised item scores
